# Response to Finch and Murray (2025)

**DOI:** 10.1017/awf.2025.8

**Published:** 2025-03-11

**Authors:** Birte L Nielsen, T Bas Rodenburg

In their Letter to the Editor, Finch and Murray (2025) criticise the way information published in an article in *Animal Welfare* (Ben-Ami *et al.* 2014) has been cited by others in the decade since its publication. Their main issue is the mention in the article of data that they consider to be unreliable, which were derived from a report that was not peer-reviewed (Ben-Ami 2009).

We welcome correspondence that will help clarify potential misleading information. However, we do not consider this to be due to oversight on behalf of the journal, as suggested by Finch and Murray (2025). There are four main reasons for this.

Firstly, the source of the data is not the article published in *Animal Welfare*, but a report produced by Ben-Ami in 2009. This is, as Finch and Murray point out, not a peer-reviewed article, but a report summarising previously collected data and available on the website of an animal advocacy group. The use of such grey literature is common in scientific publications, especially in preliminary or scoping reviews, when data are difficult to obtain otherwise.

Secondly, in Ben-Ami *et al.* (2014), the authors are quite transparent in their sources and the wording used by them did not overstate their claim: “*Therefore, the combination of the available information from the organisations and carcase-handling practices of shooters suggests that 4% or 120,000 adult kangaroos … is a conservative estimate.*” (Ben-Ami *et al.* 2014).

Finch and Murray (2025) take issue with the conclusions that 4% of kangaroos (*Macropus* and *Osphranter* spp) not shot in the head might be a lower estimate. The study by Ben-Ami (2009), that concluded that *up to* 40% of kangaroos *may have been* shot in the neck, and not the head, using indirect evidence, is only mentioned once and this information is not repeated in the article Abstract nor the Conclusion. This focus is reflected in peer-reviewed articles that cite Ben-Ami *et al.* (2014), an example being Descovich *et al.* (2015): *“Reports on carcases submitted for processing estimate that 3–4% are killed by shots to other parts of the body (Ben-Ami et al. 2014)”.* The estimates mentioned in Ben-Ami *et al.* (2014) have therefore not been presented in the *peer-reviewed literature* in a way that is ‘*deliberately misleading’*, as stated by Finch and Murray (2025).

Thirdly, the issue raised by Finch and Murray (2025) lies with the referencing of the article by Ben-Ami *et al.* (2014) in reports and enquiries that followed. However, the way in which articles published in a journal are subsequently cited is not something that any journal can control.

Fourthly, the other literature item referred to by Ben-Ami *et al.* (2014) in relation to the frequency with which kangaroos are shot in the head during commercial harvesting is a report produced by RSPCA Australia (2002), which is also not a peer-reviewed article, and not readily discoverable online. Finch and Murray (2025) argue that the data presented in RSPCA Australia (2002) are more credible than those reported by Ben-Ami in 2009, but both reports can be categorised as grey literature. A paper published in *Animal Welfare* (Hampton *et al.* 2015) reviewed the various methods used for estimating the frequency of non-fatal wounding and inaccurate shots arising from wildlife shooting programmes. This study made the case that only ante mortem data collected directly by an independent observer can be considered robust, with all forms of asynchronous post mortem examination being inherently susceptible to selection bias by shooters.

We are glad to have been given this opportunity to clarify any misunderstandings that may have arisen from this article published over a decade ago.
